# High-Valent Ni^III^ and Ni^IV^ Species Relevant to C–C and C–Heteroatom Cross-Coupling Reactions: State of the Art

**DOI:** 10.3390/molecules25051141

**Published:** 2020-03-04

**Authors:** Noel Nebra

**Affiliations:** Laboratoire Hétérochimie Fondamentale et Appliquée, Université Paul Sabatier/CNRS UMR 5069, 118 Route de Narbonne, 31062 Toulouse, France; nebra-muniz@chimie.ups-tlse.fr

**Keywords:** Ni^III^ and Ni^IV^ chemistry, cross-coupling reactions, C–H bond functionalization, trifluoromethylation, fluorination

## Abstract

Ni catalysis constitutes an active research arena with notable applications in diverse fields. By analogy with its parent element palladium, Ni catalysts provide an appealing entry to build molecular complexity via cross-coupling reactions. While Pd catalysts typically involve a M^0^/M^II^ redox scenario, in the case of Ni congeners the mechanistic elucidation becomes more challenging due to their innate properties (like enhanced reactivity, propensity to undergo single electron transformations vs. 2e^−^ redox sequences or weaker M–Ligand interaction). In recent years, mechanistic studies have demonstrated the participation of high-valent Ni^III^ and Ni^IV^ species in a plethora of cross-coupling events, thus accessing novel synthetic schemes and unprecedented transformations. This comprehensive review collects the main contributions effected within this topic, and focuses on the key role of isolated and/or spectroscopically identified Ni^III^ and Ni^IV^ complexes. Amongst other transformations, the resulting Ni^III^ and Ni^IV^ compounds have efficiently accomplished: *i)* C–C and C–heteroatom bond formation*; ii)* C–H bond functionalization; and *iii)* N–N and C–N cyclizative couplings to forge heterocycles.

## 1. Introduction

Cross-coupling reactions mediated by organometallics represent a cornerstone in the daily synthetic Chemist’s toolbox leading to complex organic scaffolds [1,2]. Since the pioneering approaches to C–C coupling in the 1960s [3], the field has experimented tremendous advances with plenty of applications in material science, drug discovery and manufacturing, or natural product synthesis [1,2]. While Pd catalysts are commonly the candidates of choice, nickel is attracting growing attention owed to its higher abundance and economic issues [4,5,6]. On the other hand, and what is considerably more relevant, the enhanced reactivity and diversity in terms of redox properties of nickel (compared to palladium) offers broad room for reaction discovery [7,8]. Since the late 1970s, Ni catalysts have been employed with success in cross-coupling reactions [9,10], with the classical Ni^0^/Ni^II^ vs. Ni^I^/Ni^III^ pathways and single electron transfer (SET) processes being commonly proposed as the most plausible redox scenarios [11,12,13]. High-valent Ni^III^ and Ni^IV^ key intermediates were recently invoked in C–C and C–heteroatom bond forming reactions, albeit their isolation or detection/characterization are typically out of reach [14,15,16,17,18,19]. First, spectroscopical identification of a Ni^III^ mediating cross-coupling reactions was performed by Kochi and co-worker as early as 1978 [20]. In this seminal work, electron paramagnetic resonance spectroscopy (EPR) and UV-Vis spectroscopy allowed one to identify the *trans*-[(PEt_3_)_2_Ni^III^(2-MeOC_6_H_4_)(Br)]^+^ species that underwent C(sp^2^)–Br coupling. While additional Ni^III^ and Ni^IV^ complexes were isolated and characterized in the following decades [21,22,23,24,25,26,27,28,29,30,31,32,33,34,35,36], investigations dealing with the isolation and characterization of Ni species in high oxidation states (+3 and +4) that are efficient in cross-coupling reactions remained latent until this century. Built on the growing interest on high-valent species, this review compiles the most remarkable pieces of work addressing the long-time elusive Ni^III^ and Ni^IV^ compounds (or analogous entities lacking Ni–C bonds) that are engaged in cross-coupling events or related transformations.

## 2. C–C Bond Forming Reactions Mediated by High-Valent Ni^III^ and Ni^IV^

Unlike Pt and Pd, the chemistry of organonickel species in the oxidation state +4 is underdeveloped. Nowadays, Pt-chemistry is mainly dominated by Pt^II^ and Pt^IV^ complexes and a plethora of stable R-Pt^IV^ species are known since the origins of organometallic chemistry. Routes to access aryl- and alkyl-Pd^IV^ complexes have appeared over the last 40 years displaying very distinct and complementary reactivity patterns to the ones observed for Pd-analogs in low oxidation states. It seems obvious that going up in the group makes the study of organometallic M^IV^ derivatives more challenging. On the other hand, Ni^IV^ intermediates are often depicted in catalytic cycles [10,11,12,13,14]. Thus, the isolation, characterization and study of Ni species in oxidation states +3 and +4 represents a major challenge in modern organometallic chemistry.

**Scheme 1:**
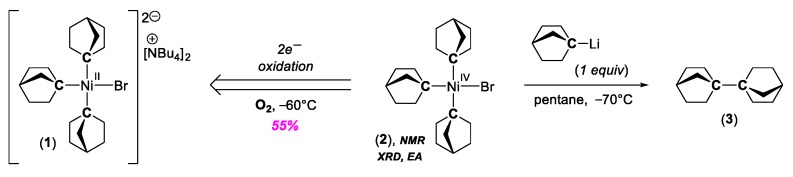
Isolation of the first Ni^IV^ complex **2** that proved active in C–C bond forming reactions. Adapted from reference [37].

Literature dealing with authenticated Ni^III^ and Ni^IV^ samples and their use in cross coupling reactions was absent until the 2000s, when the pseudotetrahedral alkyl-Ni^IV^ species **2** was reported by Dimitrov and Linden (Scheme 1) [37]. The triorganyl-Ni^IV^ complex **2** was prepared through a 2e^−^ oxidation step of the tris(1-norbornyl) precursor **1** with O_2_, and was fully characterized using nuclear magnetic resonance spectroscopy (NMR), X-ray diffraction (XRD), and elemental analysis (EA). The stability of **2** is probably given by the strong *σ*-donicity of the 1-norbornyl ligands. Remarkably, **2** resulted unstable at room temperature (r.t.) in solution and underwent elimination of dinorbornane (**3**). Alternatively, **3** was prepared upon addition of (1-norbornyl)lithium without the identification of the homoleptic tetraorganyl-Ni^IV^ intermediate.

**Scheme 2:**
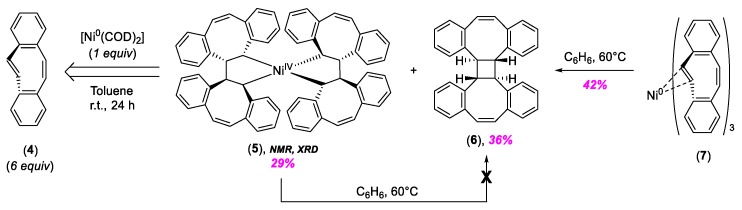
Synthesis of the homoleptic Ni^IV^ spirocycle **5** and formation of cyclobutane **6**. Adapted from reference [38].

The first homoleptic Ni^IV^ complex **5** was made via two oxidative couplings of **4** with [Ni^0^(COD)_2_] (COD = 1,5-cyclooctadiene) allowing to build the spirocyclic motif in **5** (Scheme 2) [38,39]. **5** was characterized using NMR and XRD, and proved stable upon heating or exposure to air. The enhanced stability of **5** was provided by the high shielded geometry around the Ni^IV^-center imposed by the rigidity and bulkiness of the alkyl-based chelating ligands. Interestingly, **5** comes along with the *trans*,-*trans*,*trans*-cyclobutane **6** that was formed in 36% yield. In contrast, the remarkable stability of **5** pointed to the reductive elimination (R.E.) of **6** from a low-valent nickelacycle. Alternatively, **6** was eliminated in ca. 40% yield upon mild heating of the tris((*5Z*,*11E*)-dibenzo-[*a,e*]cyclooctatetraene)nickel(0) species **7**, which was prepared from [Ni^0^(*^t^*Bu_3_P)(COD)] and **4**.

**Scheme 3:**
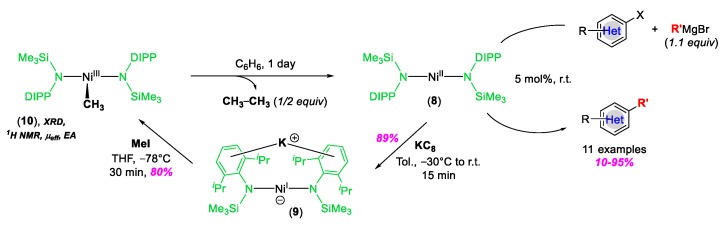
C–C coupling active Ni^III^-CH_3_ species **10** isolated and characterized by Tilley. Adapted from references [40,41].

A couple of papers detailing the isolation, characterization (XRD, ^1^H NMR, magnetic data, and EA), and reactivity of the T-shaped Ni^III^-CH_3_ complex **10** were reported by Tilley (Scheme 3) [40,41]. **10** was prepared through oxidative addition of MeI to a Ni^I^ species generated through reduction of the stable [Ni^II^(N(DIPP)SiMe_3_)_2_] precursor **8** using KC_8_. **10** is conveniently stabilized by the two rigid and bulky bis(amido) ligands, but slowly decomposes to **8** with concomitant ethane production. In addition, **8** catalyzed the coupling of aryl halides and Grignard reagents.

**Scheme 4:**
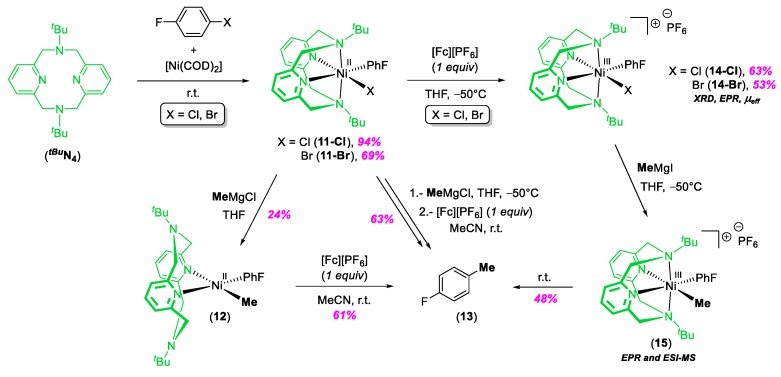
Proof of concept for the participation of aryl-Ni^III^ complexes **14-Cl**, **14-Br,** and **15** in C–C bond forming reactions with alkyl Grignard reagents. Adapted from reference [42].

Mirica has isolated the high-valent aryl-Ni^III^ compounds **14-Cl** and **14-Br** that underwent C–C coupling with alkyl Grignard reagents (Scheme 4) [42]. **14-Cl** and **14-Br** are stabilized by the *N*,*N*-di-tert-butyl-2,11-diaza[3.3](2,6)pyridinophane (***^tBu^*N_4_**) ligand and were achieved in a two-step fashion through: *i)* an initial coordination of the ***^tBu^*N_4_**ligand to Ni^0^, followed by insertion into the C–X bond and *ii)* 1e^−^ oxidation using ferrocenium hexafluorophosphate [Fc^+^][PF_6_]. XRD, EPR, paramagnetic NMR, and magnetic data for **14-Cl** and **14-Br** confirmed the octahedral coordination of Ni and pointed to the presence of a mostly Ni^III^ located unpaired electron. **14-Cl** and **14-Br** reacted with MeMgI at −50 °C to afford **15**. Notably, **15** constitutes the first di(hydrocarbyl)-Ni^III^ intermediate (identified using ESI-MS and EPR), and underwent the R.E. of 4-fluorotoluene (**13**) in moderate yield (48%). An improved yield (63%) was reached in a one-pot reaction of the aryl-Ni^II^-Br complex **11-Br** with MeMgBr and [Fc^+^][PF_6_]. The addition of 2,2,6,6-tetramethylpiperidin-1-yl)oxyl (TEMPO) as a radical trap did not affect the coupling reaction and ruled out the involvement of organic radicals. In addition, the Ni^II^ and Ni^III^ complexes **11-Cl**, **11-Br**, **14-Cl,** and **14-Br** proved suitable in Negishi and Kumada couplings.

The lack of selective methods for aromatic trifluoromethylation represents a major concern [43,44,45]. This is mainly due to: *i)* the steadily increasing demand of organofluorine materials in pharmaceutical, agrochemical, and medical applications, or in material science [46,47,48] and *ii)* the disfavored transition metal mediated aryl–CF_3_ bond formation due to the strong M–CF_3_ bonds. It is believed that the current lack of environmentally-benign [49,50,51] and industrially-suitable routes to benzotrifluorides impedes faster advance in drug discovery. The impossibility to achieve aryl–CF_3_ R.E. from a well-defined, low-valent aryl-Ni^II^-CF_3_ fragment was soon noticed independently by the groups of Vicic [52] and Grushin [53]. An elegant and certainly underexplored approach to enable the decisive R.E. step consists in the preparation of highly reactive Ni^III^ and Ni^IV^ complexes, which are commonly inaccessible. Taken all together, the use of CF_3_-groups in organometallic chemistry offers a unique balance between stability and reactivity that allows for the isolation of high-valent compounds with strong M–CF_3_ bonds, yet enables CF_3_-group transfer into strategically designed organic scaffolds. While high-valent Ni species were unidentified, Ph–CF_3_ bond formation was achieved by Sanford’s team upon 1e^−^ oxidation of well-defined [(P_2_)Ni^II^(Ph)(CF_3_)] platforms using the outer-sphere oxidant [Fc^+^][PF_6_] [54]. These studies have shown the crucial role of the ancillary diphosphine ligand on the R.E. of benzotrifluorides [54]. As might be expected, small bite angles (*β_n_* < 92°) conducted to insignificant amounts of benzotrifluoride (<10% yield), whereas bite angles ranging from 95° to 102° favored the aryl–CF_3_ coupling (up to 77% yield).

**Scheme 5:**
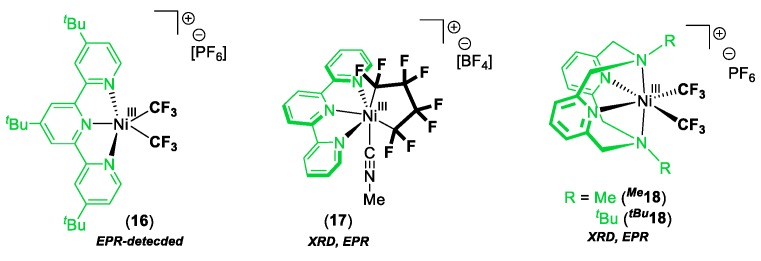
Stable Ni^III^-CF_3_ complexes **16**, ***^Me^*18,** and ***^tBu^*18** and perfluorinated nickelacycle **17** [55,56,57].

The first approach to high-valent NiCF_3_ species was reported by Vicic and co-workers [55]. They prepared and characterized in situ the cationic [(*^tBu^*terpy)Ni^III^(CF_3_)_2_][PF_6_] species **16** using low-temperature EPR (Scheme 5). Unfortunately, **16** turned out to be unstable at r.t. and yielded complex [(*^tBu^*terpy)Ni^II^(CF_3_)]^+^ via ^●^CF_3_ radical elimination. The use of a perfluorinated nickelacycle motif in **17** warranted the Ni^III^ stabilization and permitted its characterization using XRD and EPR [56]. Cyclic voltammetry of **17** displayed a low redox potential attributable to the reversible Ni^II^/Ni^III^ couple, whereas elevated redox potentials are required to overcome the Ni^III^/Ni^IV^ oxidation potentials. The first isolable Ni^III^-CF_3_ compounds ***^Me^*18** and ***^tBu^*18** were obtained in reasonable yields by Mirica’s group using highly donating tetradentate pyridinophane ligands ***^Me^*N_4_** and ***^tBu^*N_4_** [57]. ***^Me^*18** and ***^tBu^*18** were characterized using XRD, EPR, and computed by DFT (density functional theory). Besides the trifluoromethylation of the radical scavenger PBN (phenyl *N*-t-butylnitrone), no evidence was provided for the participation of **16-*^R^*18** in trifluoromethylation reactions. Instead, a ligand modification strategy was highlighted to be key in order to lower the redox potential of the Ni^III^/Ni^IV^ couple [56].

**Scheme 6:**
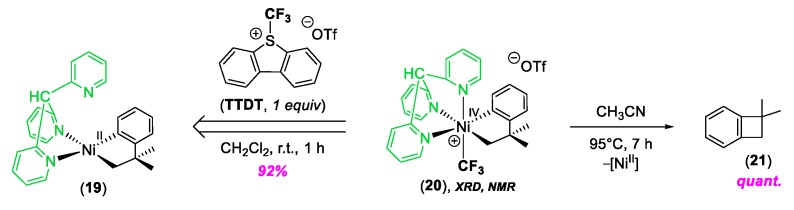
Synthesis of the Ni^IV^ complex **20**, and its use in C–C bond formation via reductive elimination (R.E.). Adapted from reference [58].

In spite of the significant number of Ni^IV^ coordination compounds that have been known since ca. 40 years ago [27,28,29,30,31,32,33,34,35,36], whether Ni^IV^ species are engaged in cross-coupling reactions or not has remained unclear until very recently. In 2015, Camasso and Sanford made a cutting-edge discovery: the isolation and complete characterization of the first Ni^IV^ compounds able to promote cross-coupling events [58,59,60]. Based on previous knowledge on related Pd^IV^-chemistry [61,62,63,64,65,66,67,68], an isolable Ni^IV^ platform was designed through the combination of three distinct strategies: *i)* the use of strongly *N*-donor, tridentate scorpionate-type ligands [i.e., tris(pyrazolyl)borate (Tp) and tris(2-pyridil)methane (Py_3_CH)]; *ii)* the presence of the Ni(cyclo-neophyl) core known to improve stability vs. R.E. and H*_β_*-elimination elementary steps; and *iii)* the employment of a *σ*-donating CF_3_-ligand (Scheme 6) [58]. Following these premises, the Umemoto reagent S-(trifluoromethyl)dibenzothiophenium triflate (TTDT) was added to **19**, and the diamagnetic Ni^IV^ complex **20** was obtained in 92% yield. The high stability of nickelacycle **20** allowed its full characterization, including XRD that confirmed the facial coordination of the Py_3_CH ligand to the octahedral Ni^IV^-center. Heated to 95 °C, **20** underwent C(sp^2^)–C(sp^3^) reductive elimination to afford quantitatively the 1,1-dimethylbenzocyclobutene **21**. This work represents the first spectroscopic support for the participation of Ni^IV^ species in cross-coupling reactions.

**Scheme 7:**
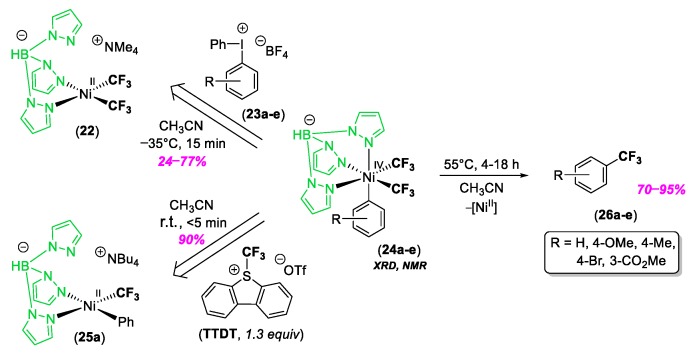
Distinct oxidation strategies to reach the Ni^IV^ species [Ni^IV^(Tp)(aryl)(CF_3_)_2_] **24a–e** from [NMe_4_][Ni^II^(Tp)(CF_3_)_2_] (**22**) and aryliodonium salts (top left) or [NBu_4_][Ni^II^(Tp)(Ph)(CF_3_)] (**25a**) and TTDT (bottom left), and R.E. of benzotrifluorides **26a–e** from **24a–e**. Adapted from reference [69].

In a following report, they performed the trifluoromethylation of aromatics from a well-defined aryl-Ni^IV^-CF_3_ fragment [69]. On this occasion, the high-valent Ni^IV^-CF_3_ complexes **24a–e** were stabilized by the tris(pyrazolyl)borate (Tp) ligand and two CF_3_-groups. **24a–e** were obtained through a 2e^−^ oxidation step of the Ni^II^-CF_3_ precursor **22** with aryliodonium salts at −35 °C in acetonitrile (Scheme 7; top left) [69]. Alternatively, the Ni^II^ to Ni^IV^ conversion can be reached using the Umemoto reagent (TTDT) as demonstrated by the high-yielding isolation of **24a** from [(Tp)Ni^II^(Ph)(CF_3_)] (**25a**; bottom left in Scheme 7) [69]. Once isolated and fully characterized, complexes **24a–e** were heated to 55 °C undergoing the elimination of the corresponding benzotrifluorides **26a–e** accompanied by [Ni^II^(Tp)_2_] and [Ni^II^(CH_3_CN)_2_(CF_3_)_2_].

Kinetic studies and Hammett plot analysis for the R.E. step enabling benzotrifluoride formation provided a *ρ* value of −0.91 indicating faster reaction rates with electron-enriched arenes [69]. This beneficial effect was attributed to the larger *trans*-effect of electron rich arenes, along with the lower kinetic barriers associated with the nucleophilic attack of the electron rich *σ*-aryl ligand to the electrophilic CF_3_-group [69]. However, the bonding analysis of complex **24a** pointed to an inverted ligand field situation [70] and the Ni^IV^ complexes **24a–e** are better described as Ni^II^ species. This effect was called the σ-noninnocence of the cationic aryl ring [70] and the release of PhCF_3_ through a redox neutral R.E. step via “*σ-noninnocence-induced masked aryl-cation transfer*”, accordingly [70].

**Scheme 8:**
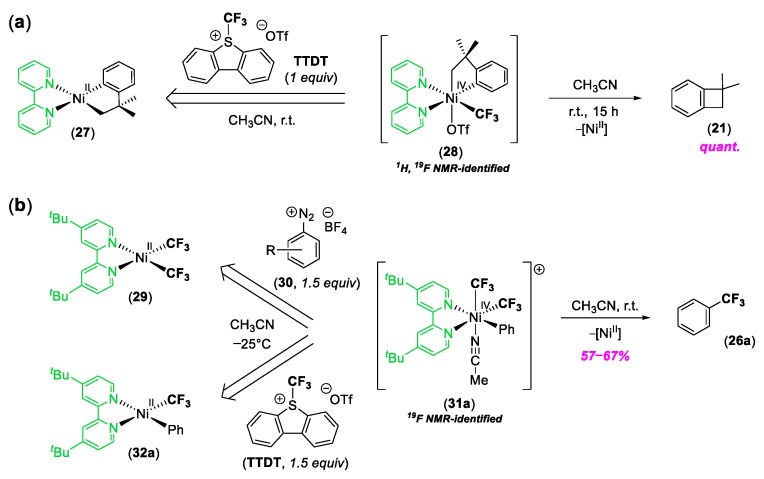
Identification of the Ni^IV^-CF_3_ key intermediates **28** and **31a** bearing bpy (**a**) or dtbpy (**b**) ligands, and subsequent R.E. steps upon warming up to r.t. Adapted from references [58,69].

The use of Py_3_CH or Tp ligands is necessary for accessing the Ni^IV^ species **20** and **24a–e** as shown by the reduced stability of analogous platforms bearing less donating bipyridine ligands (bpy or dtbpy; Scheme 8) [58,69]. As a result, the presumable Ni^IV^-CF_3_ intermediates **28** and **31a** were exclusively identified using low-temperature NMR analysis, and rapidly released 1,1-dimethylbenzocyclobutene **21** or benzotrifluoride **26a** at r.t., respectively. Replacement of the CF_3_-group by a distinct X-type ligand (i.e., halides, tosylate, or acetate) reduced the stability of Ni^IV^, thus favoring the C(sp^2^)–C(sp^3^) R.E. and hampering the detection of Ni^IV^ species.

**Scheme 9:**
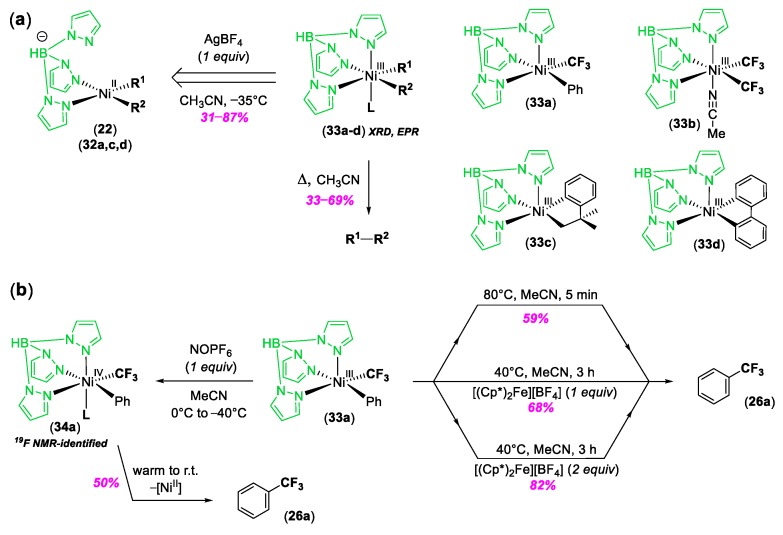
Syntheses of Ni^III^ species **33a–d** (**a**), and R.E. studies enabling Ph–CF_3_ coupling (**b**). Adapted from reference [71].

Shortly after, they evaluated the capacity of similar Ni^III^ complexes **33a**–**d** to forge diverse C–C bonds (Scheme 9) [71]. The Ni^III^ **33a–d** were achieved in variable yields from **22** and **32a,c,d** through a 1e^−^ oxidation process with AgBF_4_. The isolated Ni^III^ materials were characterized using EPR and XRD. Heated in acetonitrile, **33a,c,d** decomposed into [Ni^II^(Tp)_2_] and Ni^0^ with concomitant formation of the C–C coupled products in 33–69% yield. Remarkably, **33b** merely produced 1% of hexafluoroethane. For complex **33a** displaying the Ph-Ni^III^-CF_3_ fragment, enhanced rate and yield of benzotrifluoride **26a** was reached in the presence of oxidants, such as [(Cp*)_2_Fe][BF_4_]. This observation was attributed to the efficient quenching of the resulting Ni^I^ species generated in situ during Ph–CF_3_ formation. Detailed mechanistic investigations have demonstrated the direct R.E. from the Ni^III^ species **33a,c,d** (instead of the assumed Ni^IV^ derivatives).

**Scheme 10:**
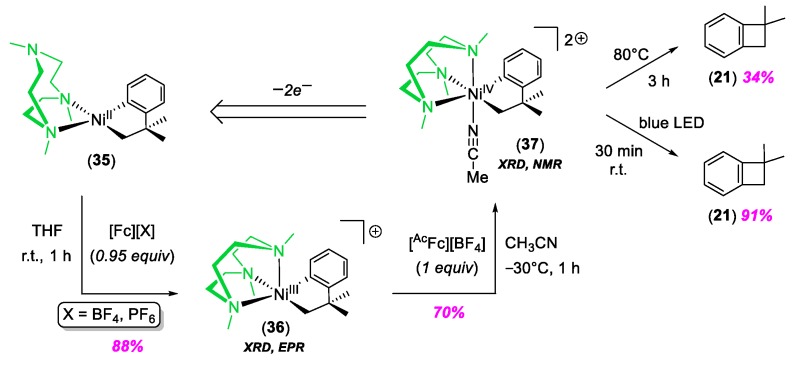
Ni^II^/Ni^III^/Ni^IV^ oxidation process starting from the Ni(cyclo-neophyl) **35** and R.E. studies from high-valent species **37** upon heating or blue LED irradiation. Adapted from reference [72].

More recently, Mirica’s group has reported the synthesis and complete characterization of the high-valent Ni^III^ and Ni^IV^ compounds **36** and **37** enabled by: *i)* facial coordination of the tridentate ligand 1,4,7-trimethyl-1,4,7-triazacyclononane (Me_3_tacn) and *ii)* the Ni(cyclo-neophyl) skeleton (Scheme 10) [72]. Complexes **36** and **37** were prepared in high yield from the Ni^II^-precursor **35** through two successive 1e^−^ oxidation steps with ferrocenium tetrafluoroborate ([Fc^+^][BF_4_]) and acetylferrocenium tetrafluoroborate ([^Ac^Fc^+^][BF_4_]). XRD studies confirmed the atom connectivity in **36** and **37** along with their square pyramidal and octahedral geometry, respectively. Heated to 80 °C, the Ni^IV^ complex **37** underwent C(sp^2^)–C(sp^3^) R.E. in modest yield while blue LED irradiation drove to almost quantitative formation of 1,1-dimethylbenzocyclobutene **21**. Interestingly, exposure of the Ni^III^ species **36** to blue LED did not improve the reaction yield. This work argues in favor of Ni^IV^ being most likely the coupling active species when dealing with dual Ni/photocatalytic approaches (instead of the commonly invoked Ni^III^ intermediates) [73,74,75,76].

**Scheme 11:**
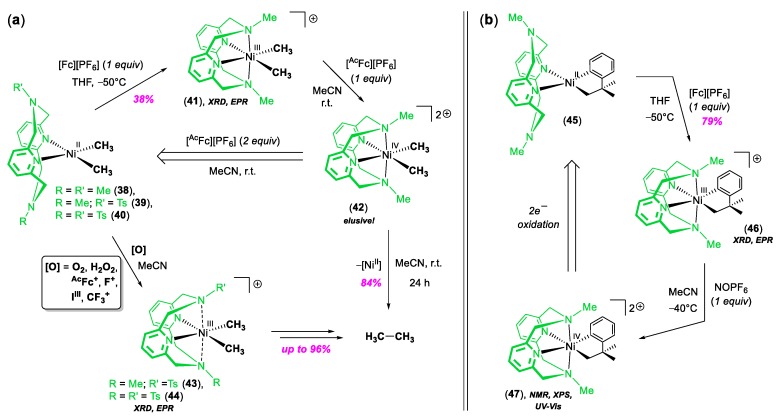
Syntheses of Ni^III^ and Ni^IV^ dialkyl complexes **41**–**44** and R.E. of ethane (**a**). Syntheses of stable Ni^III^ and Ni^IV^ cyclo-neophyl complexes **46** and **47** (**b**). Adapted from references [77,78].

The tetradentate *N*,*N*-dimethyl-2,11-diaza[3.3](2,6)pyridinophane ligand (***^Me^*N_4_**) enabled the isolation and full characterization of the first Ni^III^-dialkyl complex **41**. It was achieved from the square planar Ni^II^ precursor **38** and [Fc^+^][PF_6_] (Scheme 11a) [77], and its octahedral geometry was confirmed using XRD and EPR. **41** in acetonitrile produced ethane and methane in ca. 55% and 30%, respectively. Ethane production was improved upon addition of [^Ac^Fc^+^][PF_6_] pointing to the formation of an elusive Ni^IV^-dialkyl intermediate **42** that decomposes rapidly to Ni^II^ material and ethane via a R.E. step. In order to stabilize the high-valent species, the cyclo-neophyl group was incorporated to the Ni^II^ platform (Scheme 11b) [77]. The two-step oxidation of **45** with [Fc^+^][PF_6_] to give the Ni^III^ intermediate **46**, followed by addition of NOPF_6_ permitted the identification of the Ni^IV^ compound **47**, which was characterized using NMR and X-ray photoelectron spectrometry (XPS). The enhanced stability of the Ni^III^ and Ni^IV^ compounds **46** and **47** (vs. **43** and **44**) inhibited the R.E. and led to **21** in low yields (10% and 38%, respectively).

In a subsequent article, the same group reported the synthesis and characterization of analogous Ni^III^-dialkyl complexes **43** and **44** incorporating NMe/NTs or NTs/NTs donating groups [78]. The low donicity of the TsN-amino groups favored the formation of transient penta- or tetra-coordinated Ni^III^-dialkyl species that are more prone to eliminate ethane (Scheme 11a) [78]. Compounds **43** and **44** are easily accessible in presence of O_2_ or H_2_O_2_ and underwent selective C–C bond formation. In addition, the quantitative formation of **21** was accomplished from an elusive Ni^IV^-complex **48**, very similar to **47** but with TsN-amino groups [79].

**Scheme 12:**
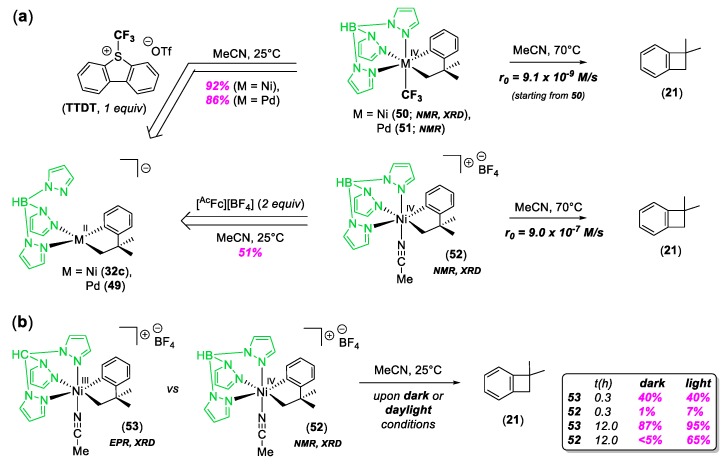
C–C bond formation as a function of the metal (Ni vs. Pd; **a**), ancillary ligand (Tp vs. Py_3_CH and CF_3_ vs. MeCN; **b** and **a**, respectively) or oxidation state at the Ni-center (+3 vs. +4; **a**). Adapted from references [80,81].

As shown earlier, Ni^III^ and Ni^IV^ complexes are engaged in C–C bond forming reactions, although limited knowledge is available concerning their comparative efficiency upon similar environments such as identical geometry, type of ligands or ligand set, and global charge. In this sense, Sanford’s group has evaluated the feasibility of C–C and C–heteroatom bond formation depending on: *i)* the nature of the transition metal (Ni vs. Pd) [80]; *ii)* the nature of the surrounding ligands (MeCN vs. CF_3_) [80]; and *iii)* the oxidation state at Ni (+3 vs. +4; see Scheme 12) [81].

Organometallic compounds **50–52** bearing a tris(pyrazolyl)borate ligand (Tp) were prepared from the corresponding salts [Q^+^][(Tp)M^II^(cyclo-neophyl)] (Q^+^ = K^+^, NMe_4_^+^; M = Ni, Pd) through selective 1e^−^ or 2e^−^ oxidation processes. Kinetic studies performed for the elimination of **21** from the M^IV^ species **50**, **51,** and **52** proved the higher stability of **50** vs. **52**, most likely due to the strong *σ*-donation of the CF_3_-group. The nature of the cationic species (Tp)-Ni^IV^ **52** and (Py_3_CH)-Ni^III^ **53** was authenticated using XRD, EPR (**53**) or NMR (**52**), and cyclic voltammetry [80,81]. Their ability to release **21** was then compared at r.t. in the dark or when exposed to daylight (Scheme 12), thus reflecting the higher activity of the (Py_3_CH)-Ni^III^ complex **53**. It provided the coupled product in 87% yield after 12 h in the dark, whereas the Ni^IV^ released negligible amounts of **21** (<10%; 300-fold slower than **53**). Exposure to daylight improved the efficiency of the Ni^IV^ complex **52** to form the C–C bond (65% in 12 h), while no remarkable effect was accounted for the Ni^III^ **53**.

**Scheme 13:**
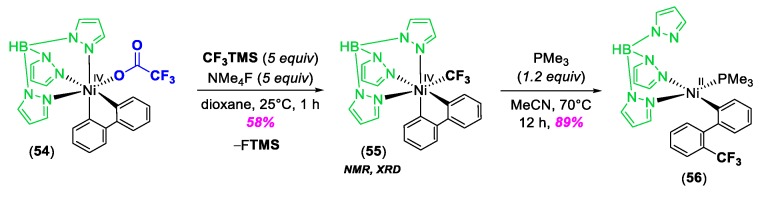
Transmetallation reaction between the Ni^IV^-O_2_CCF_3_ complex **54** and CF_3_TMS/NMe_4_F to reach **55**, and synthesis of the Ni^II^ species **56** via R.E. of aryl–CF_3_. Adapted from reference [82].

In 2017, the group of Sanford reported the transmetallation reaction between the Ni^IV^-O_2_CCF_3_ complex **54** and Ruppert’s silane in presence of [Me_4_N][F] leading to Ni^IV^-CF_3_ complex **55** (Scheme 13; the synthesis, characterization and reactivity of **54** is depicted below in Scheme 30) [82]. The Ni^IV^-CF_3_ compound **55** was conveniently characterized using NMR methods and XRD and underwent aromatic trifluoromethylation leading to **56** in ca. 90% yield upon warming at 70 °C overnight. The addition of the electron rich PMe_3_ ligand improved kinetics and yield of aryl–CF_3_ production.

**Scheme 14:**
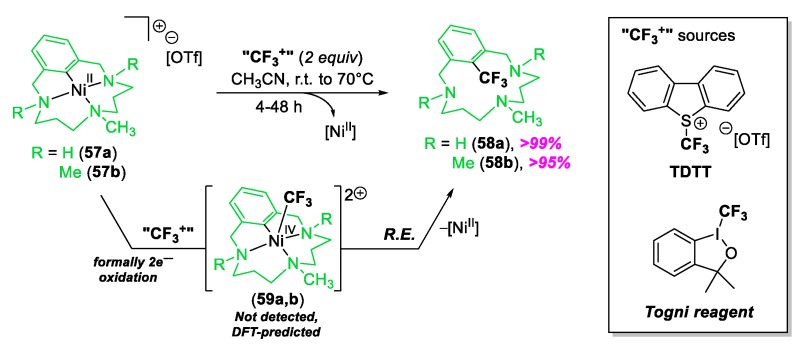
Oxidative trifluoromethylation of N_3_C_arom_ ligands mediated by Ni. C–CF_3_ bond formation from the elusive (N_3_C_arom_)Ni^IV^ intermediates **59a,b**. Adapted from reference [83].

The same year, Ribas and co-workers reported the quantitative trifluoromethylation of triaza-macrocycles bearing an aromatic ring [83]. The reaction involves two independent steps namely: *i)* an aryl-Ni^II^ bond formation to reach **57a,b** via aryl–Br oxidative addition to [Ni^0^(COD)_2_], or alternatively, C–H bond nickelation using [Ni^II^(NO_3_)_2_]•6(H_2_O); and *ii)* the oxidative trifluoromethylation of the macrocyclic scaffold upon addition of the Umemoto or Togni reagents (Scheme 14) [83]. The authors proposed a Ni^II^ to Ni^IV^ oxidation step prior to R.E. of the coupled products **58a,b** through an initial SET process to form a (N_3_C_arom_)Ni^III^ intermediate and a ^●^CF_3_ radical that subsequently recombine with each other to build the (N_3_C_arom_)Ni^IV^ species **59a,b**.

**Scheme 15:**
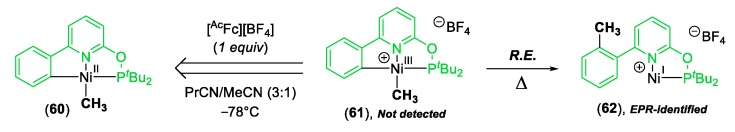
Aryl–CH_3_ coupling from an assumed cationic [(PNC_arom_)Ni^III^(CH_3_)]^+^ pincer complex **61**. Adapted from reference [84].

Klein, van der Vlugt and co-workers performed the 1e^−^ oxidation of the Ni^II^-CH_3_ complex **60** upon addition of [^Ac^Fc^+^][BF_4_] to yield the transient Ni^III^-CH_3_ species **61** bearing an aryl-pyridine-phosphine (PNC_arom_) pincer ligand (Scheme 15) [84]. Reaction monitoring using ^1^H NMR indicated the formation of the tolyl fragment and the release of the Ni^I^ complex **62**, which was identified using EPR.

**Scheme 16:**
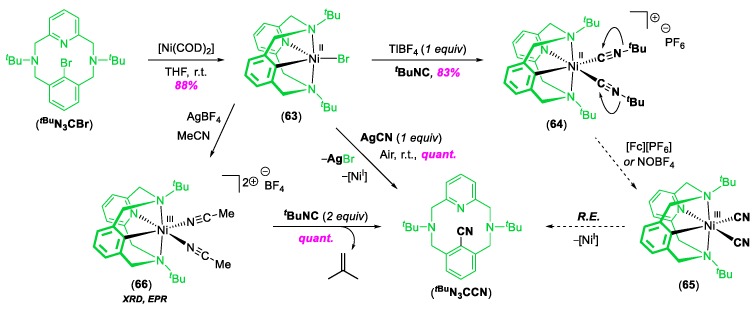
Ni^III^-mediated aromatic cyanation using *^t^*^Bu^N-containing pyridinophane scaffolds. Adapted from reference [85].

A ligand design strategy allowed the development of the oxidative cyanation of aromatic rings enabled by a Ni^III^-CN key intermediate **65** (Scheme 16) [85]. Insertion of [Ni^0^(COD)_2_] into the triaza-macrocycle ***^t^*^Bu^N_3_CBr** provides [(*^t^*^Bu^N_3_C)Ni^II^(Br)] (**63**). **63** further reacts with TlPF_6_ and *^t^*BuNC affording the highly stable bis(isocyanide) compound [(*^t^*^Bu^N_3_C)Ni^II^(*^t^*BuNC)_2_][PF_6_] (**64**). In contrast, oxidative treatment of **64** with [Fc^+^][PF_6_] or NOBF_4_ yielded the cyanide-containing macrocycle ***^t^*^Bu^N_3_CCN** instantaneously. The Ni^III^-mediated N–*^t^*Bu heterolytic bond scission to give the Ni^III^-CN species and isobutylene was proposed in line with: *i)* the high stability of the Ni^II^-CN*^t^*Bu species **64**; and *ii*) the quantitative yield of ***^t^*^Bu^N_3_CCN** attained upon addition of AgCN to [(*^t^*^Bu^N_3_C)Ni^II^(Br)] (**63**) [86]. The bis(acetonitrile) Ni^III^ complex (**66**) was conveniently characterized using XRD and EPR, and was reacted with *^t^*BuNC resulting in the liberation of ***^t^*^Bu^N_3_CCN**. Monitoring the coupling reaction illustrated the simultaneous consumption of **66** and the formation of ***^t^*^Bu^N_3_CCN**, thus suggesting the participation of Ni^III^ species in the N–*^t^*Bu bond breaking/aryl–CN bond forming sequence.

**Scheme 17:**
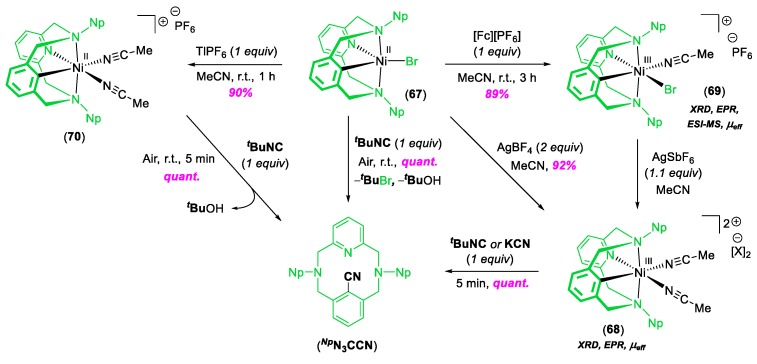
Ni^III^-mediated aromatic cyanation using *^Np^*N-containing pyridinophane scaffolds. Adapted from references [87,88].

The nature of the pending RN-amino groups located at the apical positions drastically affects the stability and reactivity of the Ni^III^ species, whereas modification of the aryl moiety does not impact reactivity significantly [87]. Complex **67** undergoes the full cyanation of the aromatic ring upon addition of *^t^*BuNC in air (Scheme 17) [87]. Alternatively, ***^t^*^Bu^N_3_CCN** can be forged through initial bromide abstraction and exposure to air. These aerobic cyanations occur at r.t. within 5 min and imply the transient formation of Ni^III^ intermediates, as convincingly proved through the reactivity of the isolated Ni^III^ **68** towards *^t^*BuNC that afforded ***^t^*^Bu^N_3_CCN** in quantitative yield. High-yielding syntheses of Ni^III^ species **69** and **68** were accomplished via 1e^−^ oxidation either with [Fc^+^][PF_6_] or AgBF_4_ [88]. A distorted octahedral coordination of Ni and the presence of an unpaired electron in **68** and **69** was confirmed using XRD, EPR, and magnetic measurements [88].

**Scheme 18:**
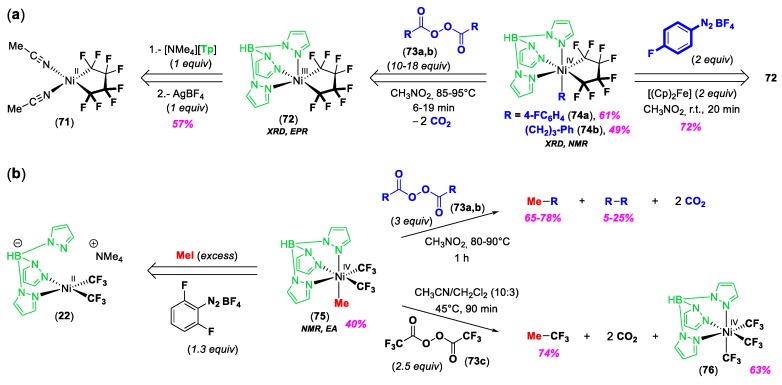
Ni^III^ to Ni^IV^ oxidation mediated by carbon-centered radicals (**a**), and C–C coupling from Ni^IV^-CH_3_ **75** and diacyl peroxides **73a**–**c**. Adapted from reference [89].

The viability of Ni^II^/Ni^III^/Ni^IV^ oxidation sequences involving successive SET processes has been investigated recently (Scheme 18) [89]. The isolation and characterization of Ni^IV^ metallacycles **74a,b** was accomplished by controlled release of alkyl and aryl radicals upon heating of the Ni^III^ precursor **72** in presence of the corresponding diacyl peroxide **73a,b** (Scheme 18a) [89]. An appropriate choice of perfluorinated ligand is necessary in order to enhance the Ni^IV^ stability, and negligible amount of Ni^IV^-CF_3_ material was formed when starting from the parent compound **33b**. In-depth mechanistic studies proved the release of free ^●^R radicals and their subsequent recombination with **72** to build the Ni^IV^. In contrast, **74a** can be synthesized in high yield from **72** and aryl radicals (aryldiazonium salts combined with ferrocene; Scheme 18a) [89].

On the other hand, the diamagnetic Ni^IV^-CH_3_ species **75** was prepared in moderate yield from the Ni^II^-CF_3_ complex **22** and MeI, and was characterized using multinuclear NMR and EA. Most remarkably, **75** reacted with carbon-based ^●^R radicals (generated from diacyl peroxides **73a–c**) and underwent R–CH_3_ bond formation (65–78% yield; Scheme 18b) [89]. The operative radical substitution (*S_H_2*) pathway and the absence of free ^●^CH_3_ radicals were concluded according to: *i)* the observed product distribution; *ii)* the lack of ethane formation; and *iii)* the marginal influence of *β*-nitrostyrene.

**Scheme 19:**
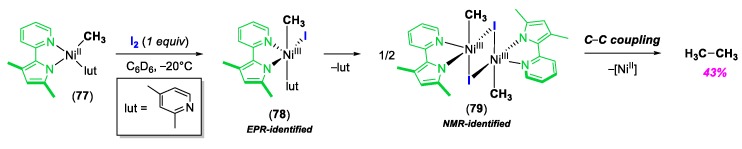
Bimolecular C–C coupling enabled by the Ni^III^ species **79**. Adapted from reference [90].

Thorough mechanistic studies including one- and two-dimensional ^1^H NMR experiments, deuterium labelling, kinetic and crossover experiments, and EPR monitoring was performed by Diao and co-workers to prove the involvement of the [(py-pyrr)Ni^III^(I)(CH_3_)]_2_ species **79** in C(sp^3^)–C(sp^3^) bond formation (Scheme 19) [90]. The mixture of high-valent isomers **79** was achieved upon 1e^−^ oxidation of the Ni^II^-precursor **77** with I_2_ at low temperature. The I-bridged binuclear complex **79** is diamagnetic due to antiferromagnetic coupling between the two low-spin Ni^III^ centers. **79** was characterized using ^1^H and ^13^C NMR at low temperature and its structure was attributed according to DFT calculations and experimental observations. The capacity of **79** to mediate C(sp^3^)–C(sp^3^) couplings was demonstrated by simultaneous ethane formation and consumption of the Ni^III^-CH_3_ **79** (Scheme 19) [90]. The bimolecular pathway was evidenced by crossover experiments, the observed dependence of the [CH_3_–CH_3_]/[CH_3_–I] ratio on [77], and the first order dependence in [79]. The synthesis of the Ni^III^-CH_3_ isomers in **79** and subsequent ethane formation involves: *i)* 1e^−^ oxidation from Ni^II^-CH_3_ **77** to the square pyramidal Ni^III^-CH_3_ monomer **78** (EPR-identified); *ii)* dimerization of **78** leading to diamagnetic Ni^III^ material **79** upon lutidine dissociation; and *iii)* C–C bond formation with concomitant reduction of **79** to Ni^II^.

## 3. C–Heteroatom Bond Formation Mediated by High-Valent Ni^III^ and Ni^IV^

Amongst all types of cross-coupling reactions, C–heteroatom bond forming reactions constitute a useful and reliable tool in organic synthesis leading to relevant heterocyclic scaffolds and aryl derivatives such as phenols or anilines. In marked contrast to Pd-catalyzed cross-coupling reactions that operate through a M^0^/M^II^ catalytic loop, Ni^III^ species are commonly accepted as key intermediates in C–heteroatom couplings since the early discoveries made by Kochi and co-worker in 1978 [20]. In this sense, seminal work by the group of Hillhouse has provided additional insights for the participation of cyclometallated Ni^III^ compounds in the challenging C–heteroatom bond formation step that typically hampers catalytic turnover. Even though the authors failed to characterize the high-valent Ni^III^-intermediates, they have illustrated the potential utility of high-valent Ni^III^ species in C–heteroatom couplings giving rise to pyrrolidine [91,92], 3,4-dihydrocoumarin [92,93], or aziridine [94] scaffolds.

**Scheme 20:**
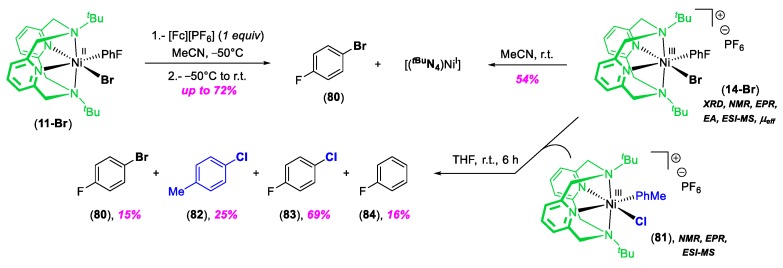
Proof of concept for the participation of well-defined aryl-Ni^III^-X intermediates **14-Br** and **81** in C(sp^2^)–X bond forming reactions. Adapted from reference [42].

The reactivity of the aryl-Ni^III^-X complexes **14-Cl** and **14-Br** towards alkyl Grignard and alkyl Zn derivatives was reported in 2014 (Scheme 4) [42]. In absence of an alkyl-type organometallic partner, the high-valent species **14-Cl** and **14-Br** underwent C(sp^2^)–Cl and C(sp^2^)–Br bond forming reactions upon warming up to r.t. (Scheme 20) [42]. Alternatively, the addition of [Fc^+^][PF_6_] to the aryl-Ni^II^ complexes **11-Cl** and **11-Br** in acetonitrile at −50 °C and ensuing exposure to r.t. leads to the corresponding aryl halides in up to 72% yield. The stirring of Ni^III^ complexes **14-Br** and **81** in equimolar amounts at r.t. yielded the C(sp^2^)–X coupled products **80**, **82–84** due to Ni^III^-X bond dissociation and subsequent C–halide bond formation. This work has provided the first spectroscopic evidence for the Ni^IV^-involvement in C–heteroatom coupling.

**Scheme 21:**
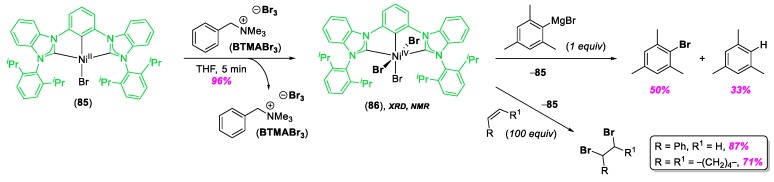
C–Br couplings enabled by Ni^IV^Br_3_ species **86**. Adapted from reference [95].

The addition of bromine (Br_2_) or its safer substitute [BnNMe_3_][Br_3_] to **85** led to the nearly quantitative isolation of the Ni^IV^Br_3_ compound **86** bearing a bis-carbene pincer platform (^DIPP^CCC; Scheme 21) [95]. XRD and ^1^H NMR confirmed the atom connectivity and the octahedral coordination in **86**. The 2e^−^ reduction process in presence of organic substrates such as olefins or (mesityl)MgBr restores the Ni^II^-Br **85** and yields the brominated products (Scheme 21).

**Scheme 22:**
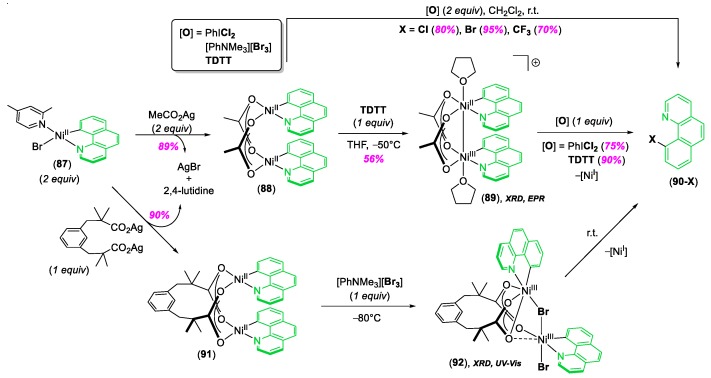
C(sp^2^)–X couplings enabled by high-valent Ni^III^–X–Ni^III^ homobimetallic complexes. Adapted from reference [99].

In analogy to Pd^III^-chemistry [96,97,98], the role of intermetallic interactions when dealing with cross-coupling reactions and high-valent Ni homobimetallics has been investigated (Scheme 22) [99]. Thus, the unprecedented Ni platforms **89** and **92** containing the benzo[*h*]quinoline ligands were isolated and characterized. XRD, EPR, and DFT analyses on the homobimetallic complex **89** pointed to: *i)* the presence of a binuclear Ni complex with a Ni–Ni bond order of ½; *ii)* an average oxidation state of +2.5 for each Ni-center; and *iii)* the stabilization of the electrodeficient [Ni_2_]^5+^ core by apical coordination of THF ligands. Addition of TDTT or PhICl_2_ to **89** afforded the coupled products in 90% and 75% yield, respectively. In contrast, the addition of bromide anions to **89** resulted unfruitful. The 2e^−^ oxidation of the binuclear Ni^II^ complex **91** with [PhNMe_3_][Br_3_] gave rise to the Ni^III^–Br–Ni^III^ species **92**, which was characterized using XRD thereby proving the lack of a Ni–Ni bond. Warming up to r.t., the binuclear Ni^III^–Br–Ni^III^ complex **92** underwent C(sp^2^)–Br bond formation and yielded **90**-**Br** (Scheme 22) [99]. The high activity of the assumed Ni^III^–X–Ni^III^ species coupled to the inactivity of the mixed-valence complex **89** seems to indicate that the C(sp^2^)–X coupling occurs at each Ni^III^-center only in absence of any Ni–Ni interaction.

**Scheme 23:**
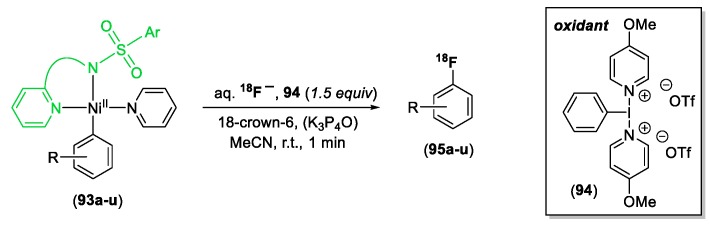
C(sp^2^)–^18^F coupling enabled by the Ni^II^-platform **93a-u**, aqueous ^18^F^−^ and **94**. Adapted from references [100,101].

The selective incorporation of fluorine atoms to organic scaffolds is highly desirable due to: *i)* the unfavored R–F coupling from a well-defined R–M–F fragment; and *ii)* the importance of organofluorine chemistry in industry [44,46,47,48]. An impressive strategy to build C(sp^2^)–^18^F bonds mediated by aryl-Ni^II^ precursors **93a–u**, the iodine(III)-based oxidant **94** and aqueous ^18^F^−^ has been recently developed by Ritter and colleagues (Scheme 23) [100,101].

**Scheme 24:**
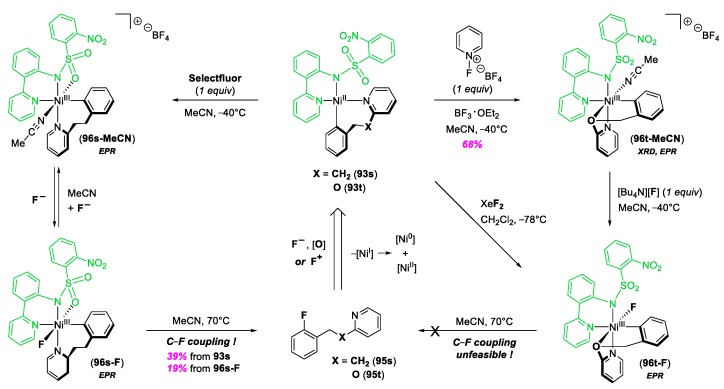
Aromatic fluorination enabled by Ni^III^–F intermediates. Adapted from reference [102].

A common hallmark in the Ni^II^-platform **93a–u** resides in the sulfonamide moiety included in the bidentate ancillary ligand, a mandatory requirement for the C(sp^2^)–^18^F bond forming reaction to proceed. While no high-valent Ni-species were initially detected, in situ EPR characterization of the key aryl-Ni^III^ species **96s,t-MeCN,** and **96s,t-F** was carried out in a subsequent article (Scheme 24) [102]. Enhanced stability of Ni^III^ was achieved upon the use of the more rigid Ni^II^ platforms **93s,t** bearing the chelating *σ*-aryl-pyridine ligands. This strategy proved right, and the sulfonamide-stabilized key intermediates **96s-MeCN** and **96s-F** underwent C(sp^2^)–^18^F coupling upon mild heating. In sharp contrast, the constrained geometry displayed by the parent aryl-Ni^III^ complexes **96t-MeCN** and **96t-F** prevented the aromatic fluorination.

**Scheme 25:**
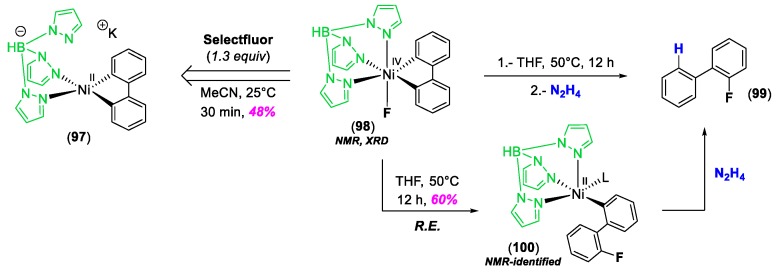
Aromatic fluorination enabled by the Ni^IV^–F complex **98**. Adapted from reference [103].

A Ni^IV^–F compound which enables aromatic fluorination has been recently reported (Scheme 25) [103]. The Ni^II^ complex **97** bearing the potentially tridentate tris(pyrazolyl)borate ligand (Tp) was reacted with selectfluor to afford the diamagnetic aryl-Ni^IV^–F species **98** in ca. 50% yield. The Ni^IV^ nature of **98** was authenticated using NMR and XRD. Upon mild heating, **98** yielded **100** that further reacts with N_2_H_4_ to cleave the Ni–aryl bond producing the fluorobiphenyl **99**. An analogous Ni^IV^–F stabilized by 2,2′-bipyridine (bpy) ligand was identified using ^19^F NMR as well before quantitative C(sp^2^)–F bond formation. DFT-calculations supported a concerted C(sp^2^)–F reductive elimination pathway with lower activation barriers for the [(bpy)Ni^IV^(aryl)(F)]^+^ key intermediate.

**Scheme 26:**
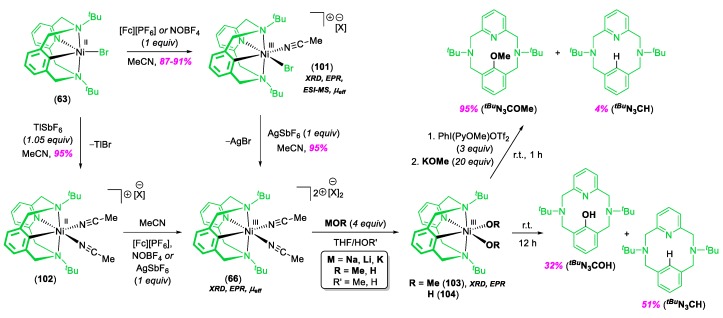
Ni^III^-OR complexes **103** and **104**, and their decomposition to build C(sp^2^)–OR bonds. Adapted from reference [104].

After the discovery of C(sp^2^)–X coupling (X = Br or Cl) mediated by isolated Ni^III^ complexes the participation of Ni^III^ species in C(sp^2^)–O bond formation was studied (Scheme 26) [104]. The salts **101** and **66** were synthesized and fully characterized using XRD, EPR, and magnetic data. The Ni^III^–OR intermediates **103** and **104** were obtained by adding metallic alkoxides or hydroxides to **66** in alcoholic or aqueous media, respectively. Attempts to isolate **103** and **104** resulted unfruitful due to their high instability. Nevertheless, the structure of **103** was corroborated by: *i)* low-resolution XRD that confirmed its octahedral geometry; and *ii)* the large gave value of 2.192 obtained by EPR (vs. 2.145 and 2.125 for **101** and **66**, respectively) that substantiated the coordination of the stronger *σ*- and *π*-donating methoxide ligands.

The Ni^III^-OMe **103** decomposes in THF at r.t. to ***^t^*^Bu^N_3_COMe** and ***^t^*^Bu^N_3_CH** in ca. 1:1 ratio (Scheme 26) [104]. The addition of an exogenous oxidant (PhI(PyOMe)_2_OTf_2_) and additional KOMe improved the selectivity towards *^t^*^Bu^N_3_C–OMe bond formation. The parent Ni^III^-OH compound **106** displays a similar EPR pattern to **103** and decomposes rapidly to afford ***^t^*^Bu^N_3_COMe** (32%) and ***^t^*^Bu^N_3_CH** (51%). The aryl–OR coupling takes place via a disproportionation reaction of **103** and **104** to generate an elusive [(*^t^*BuN_3_C)Ni^IV^(OR)_3_] species and subsequent R.E step.

**Scheme 27:**
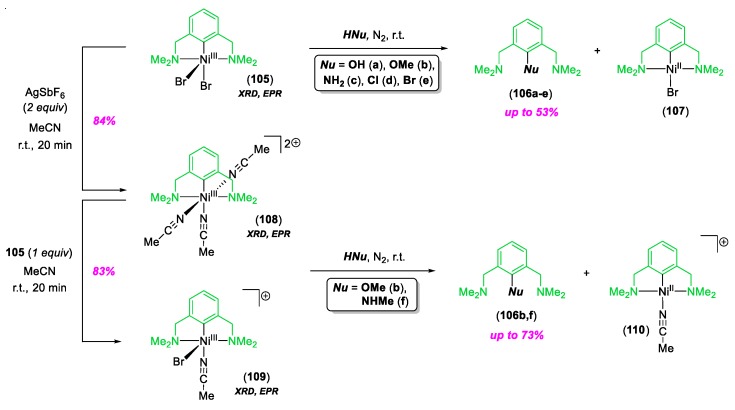
Ni^III^-mediated C–O and C–N bond formation assisted by an NC_arom_N-pincer ligand. Adapted from references [107,108].

Using the fully characterized Ni^III^Br_2_ complex **105** (Scheme 27) [105,106], C_ipso_–heteroatom bond formation with several nucleophiles (water, methanol, ammonia, or hydrobromic acid) has been studied [107]. Metal–Ligand cooperation was invoked to cleave the H–Nu bond. Thus, a Me_2_N-sidearm decoordinates and attacks the proton to create the aryl-Ni^III^-Nu moiety that is required to make possible the C(sp^2^)–Nu coupling. Nevertheless, the coupled products are reached at best in ca. 50%. The structures of Ni^III^ derivatives **109** and **108**, prepared through halogen abstraction with AgSbF_6_, were elucidated using XRD and EPR (Scheme 27) [108]. Both Ni^III^ species **108** and **109** underwent C(sp^2^)–O and C(sp^2^)–N couplings at r.t. with concomitant formation of **110**. The low yields were attributed in part to unwanted side-reactions namely: *i)* the generation of Ni^II^ species **107** and **110**; *ii)* protodemetallation; or *iii)* C(sp^2^)–OH coupling with adventitious water.

**Scheme 28:**
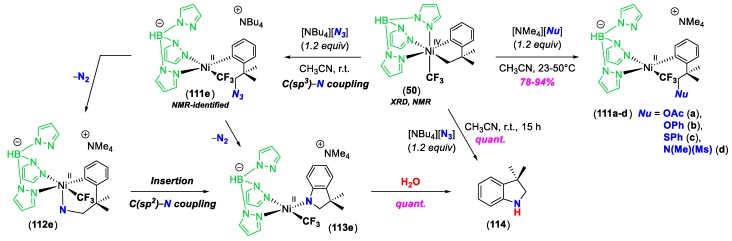
Proof of concept for the involvement of Ni^IV^ in C–heteroatom bond formation via R.E. Adapted from references [58,80].

As mentioned before, the Ni^IV^-CF_3_ compounds **20** and **50** bearing tridentate scorpionate-type ligands (Py_3_CH or Tp) underwent C(sp^3^)–C(sp^2^) cyclization (Scheme 6 and Scheme 12, respectively) [58]. Addition of heteroatom-based nucleophiles (i.e., alkoxides or amides) afforded the C(sp^3^)–heteroatom coupled complexes **111a–d** (78–94% yield; Scheme 28) [58,80]. Swain–Scott nucleophilicity parameters and kinetic studies pointed to a S_N_2-type mechanistic pathway proceeding through nucleophilic attack of the exogenous heteroatom-based nucleophile into the Ni^IV^–C(sp^3^) bond. Comparative kinetic studies with analogous (Tp)Pd^IV^CF_3_ complexes proved the higher propensity of the Ni^IV^-platform towards C(sp^3^)–OAc coupling [80]. Intriguingly, the addition of [NBu_4_][N_3_] to **50** produced 3,3′-dimethylindoline (**114**) via double C–N bond forming reaction [58,80]. The formation of **114** occurs as follow: *i)* first C(sp^3^)–N coupling giving rise to the diamagnetic Ni^II^-CF_3_ complex **111e**; followed by *ii)* N_2_-elimination and indoline-ring formation leading to the Ni^II^-CF_3_ **113e**; and *iii)* protodemetallation step with adventitious water.

**Scheme 29:**
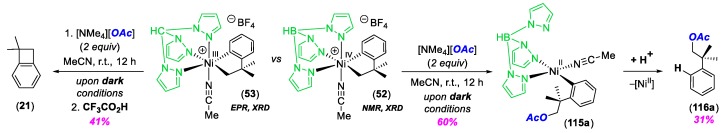
Reactivity of cationic Ni^III^ and Ni^IV^ platforms **52** and **53** vs. [NBu_4_][OAc]. Adapted from references [80,81].

The reactivity of closely related high-valent species **52** and **53** towards tetramethylammonium acetate was recently addressed as well (Scheme 29) [80,81]. As depicted in Scheme 12, the C(sp^3^)–C(sp^2^) cyclization to yield **21** proceeds more easily from the Ni^III^ **53** in dark conditions or exposed to daylight. Accordingly, addition of acetate as an exogenous nucleophile preferentially led to **21** in ca. 40% yield. In sharp contrast, the Ni^IV^ complex **52** underwent selective C(sp^3^)–OAc bond formation. Protonolysis with trifluoroacetic acid (TFA) delivered **116a**. The very distinct reactivity of **52** vs. **53** was attributed to the significantly enhanced electrophilicity of the Ni–C(sp^3^) bond in the Ni^IV^ platform **52** vs. **53**, thus favoring the nucleophilic attack via outer-sphere S_N_2 pathway.

**Scheme 30:**
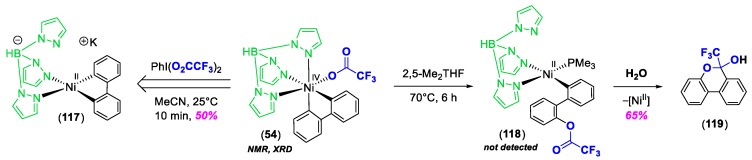
Synthesis of Ni^IV^–O_2_CCF_3_ complex **54** and C–O coupling to build the hemiketal **119**. Adapted from reference [82].

The Ni^IV^–O_2_CCF_3_ species **54** was synthesized through 2e^−^ oxidation of the anionic Ni^II^ complex **117** using bis(trifluoroacetoxy)iodobenzene (PhI(OTFA)_2_; Scheme 30) [82]. **54** was fully characterized (NMR, XRD, and EA) and proved stable in solution at −35 °C. In contrast, it slowly underwent C(sp^2^)–O bond formation in 2,5-dimethyltetrahydrofuran at r.t. Warming **54** up to 70 °C for 6 h led to heterocycle **119** through initial C–O bond formation giving rise to **118** followed by cyclization reaction and hydrolysis with moisture.

**Scheme 31:**
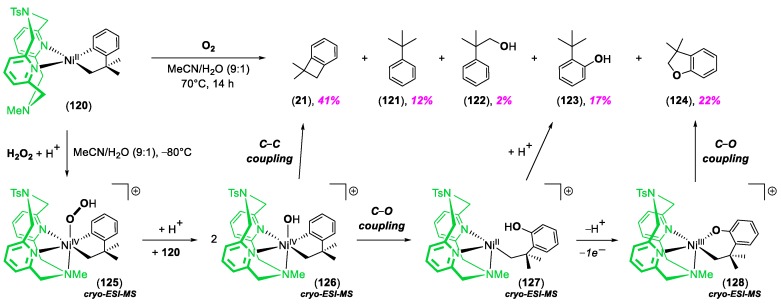
C–O bond formation enabled by high-valent Ni species **125**, **126,** and **128** generated from the Ni^II^ precursor **120** and O_2_ or H_2_O_2_ as oxidants. Adapted from reference [79].

Mirica and colleagues have explored C–O bond formations using O_2_ or H_2_O_2_ as additives. These green and environmentally friendly oxidants allow to convert the nickelacycle **120** to high-valent Ni complexes, eventually acting as coupling partners (Scheme 31) [79]. NMR and GC-MS monitoring for the oxidation of **120** with O_2_ permitted the quantification of the reaction products **121** (protonolysis), **21** and **122-124** (C–C and C–O couplings, respectively). The Ni^IV^-hydroperoxo **125**, the Ni^IV^-hydroxo **126** and the hydroxylated Ni^III^(cyclo-neophyl) species **128** were identified using cryo-ESI-MS that suggested their participation in the C–O bond forming reactions.

## 4. C–H Bond Activation and/or Functionalization Enabled by High-Valent Ni^III^ and Ni^IV^

Direct C–H functionalization is preferred over the use of pre-functionalized substrates in view of reduced-waste production, at the same time the less activated C–H bonds makes these reactions more challenging [109,110,111]. Electrophilic substitution, which works pretty smart when using Pt^II^ and Pd^II^ catalysts fails for Ni^II^ and new approaches have focused on either Ni^I^ or high-valent Ni^III^ or Ni^IV^ for this C–H activation. Remarkable efforts have been made in recent years on Ni-catalyzed C–H bond activation and functionalization enabled by directing groups, commonly requiring the use of sacrificial oxidants [112,113]. From a mechanistic point of view, most recent work by Chatani [14,15] and Ackermann [16,17] suggested a first C–H bond activation step enabling the formation of stable cyclometallated Ni^II^ species followed by a C–C or C–heteroatom coupling step from an in situ generated high-valent Ni species. Thus, there is current mechanistic debate dealing with the involvement of either Ni^I^/Ni^III^ or Ni^II^/Ni^IV^ redox scenarios; different pathways were found viable by both computational and experimental methods [114,115,116,117,118,119]. However, reports elaborating on the isolation/identification of high-valent Ni species participating in C–H bond functionalization are very rare [120].

**Scheme 32:**
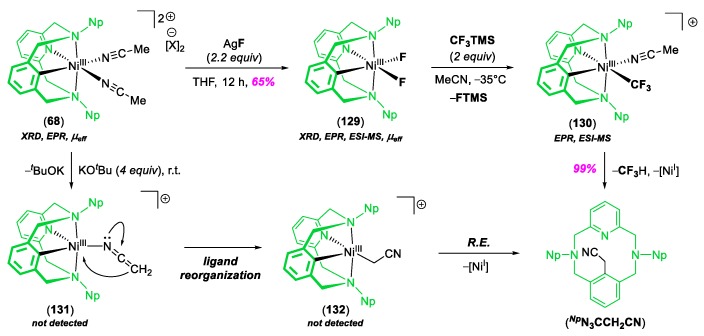
C–H bond cyanomethylation promoted by Ni^III^. Adapted from reference [88].

In 2016, the involvement of Ni^III^ species in oxidative C–H bond activation and functionalization was demonstrated (Scheme 32) [88]. A family of stable Ni^III^ complexes bearing the tetradentate pyridinophane ligand (***^Np^*N_3_C**) were prepared and characterized. The Ni^III^ complexes **130** and **68** underwent aromatic cyanoalkylation assisted by an intramolecular (CF_3_ ligand in **130**) or external (KO*^t^*Bu in **68**) base that cleaves the C(sp^3^)–H bond. EPR monitoring and radical trap experiments pointed to the intermediacy of Ni^III^ species **131** and **132**. These transient Ni^III^ species are formed through: *i)* deprotonation of MeCN to afford the ketenimine moiety in **131**; and *ii)* ketenimine redistribution giving access to **132**. The cyanoalkylated product ***^Np^*N_3_CCH_2_CN** is finally released via reductive elimination from **132**.

**Scheme 33:**
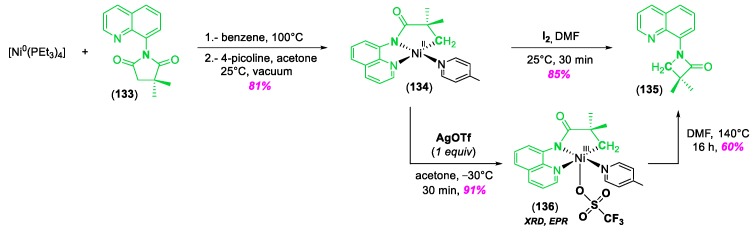
Access to cyclometallated *σ*-alkyl Ni^III^ complex **136** and synthesis of the *β*-lactam **135**. Adapted from reference [122].

Chatani carried out oxidative C–heteroatom couplings of quinoline-substituted amides catalyzed by Ni and suggested the participation of either Ni^III^ or Ni^IV^ intermediates [121]. Shortly after, Sanford and co-workers successfully prepared cyclometallated *σ*-alkyl and *σ*-aryl Ni^II^ complexes that were evaluated in C(sp^3^)–N or C(sp^2^)–I couplings upon oxidation with molecular I_2_ [122]. High-valent *σ*-aryl-Ni^III^ species are reachable upon 1e^−^ oxidation with silver salts, but failed to achieve the C(sp^2^)–I coupling. On the contrary, the *σ*-alkyl Ni^III^ complex **136** was isolated in 91% yield, was fully characterized using NMR and XRD, and gave rise to the *β*-lactam **135** via C(sp^3^)–N cyclizative coupling (Scheme 33) [122]. Nevertheless, harsh conditions were required and **136** resulted inactive under catalytic conditions.

**Scheme 34:**
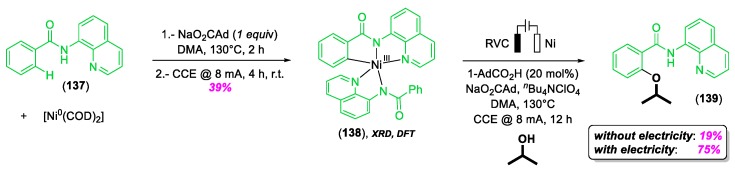
Synthesis of the *σ*-aryl-Ni^III^ intermediate **138**, and **138**-enabled C(sp^2^)–O*^i^*Pr bond formation in the presence or absence of electricity. Adapted from reference [123].

C(sp^2^)–H functionalization enabled by high-valent Ni compounds has been reported by Ackermann (Scheme 34) [123]. They carried out a nickellaelectro-catalyzed C(sp^2^)–H bond alkoxylation of aminoquinoline-based substrates such as **137**, and provided support for: *i)* the Ni^III^-mediated C(sp^2^)–OR coupling in presence or absence of electricity; and *ii)* the catalytic performance of the cyclometallated *σ*-aryl Ni^III^ **138**. This Ni^III^ **138** was obtained in 39% yield upon electrolytic oxidation from [Ni^0^(COD)_2_] and **137**, and was characterized using XRD and cyclic voltammetry (easy over-oxidation at 0.50 V vs. Fc^0/+^).

Mechanistic studies consisting of radical trap and competition experiments, evaluation of kinetic isotope effects and DFT-analysis pointed to: *i)* facile C(sp^2^)–H bond scission; *ii)* involvement of radicals; and *iii)* C(sp^2^)–OR coupling occurring from a transient formally *σ*-aryl Ni^IV^ key intermediate, which is better described as a ligand centered radical Ni^III^ species [123]. In short, the Ni^III^ complex **138** constitutes the first isolated high-valent Ni species enabling C(sp^2^)–H functionalization under stoichiometric and catalytic conditions [14,15,16,17,18,19,114,115,116,117,118,119].

**Scheme 35:**
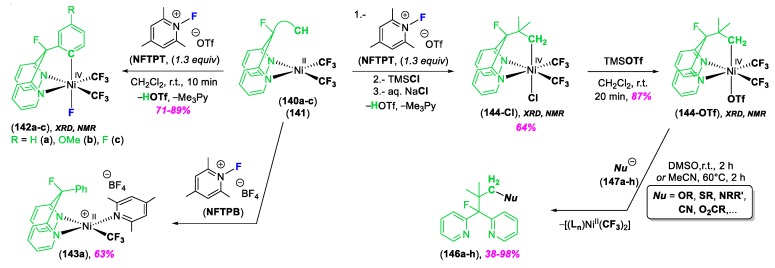
C–H bond breaking/C–Ni^IV^ bond forming sequence via Ni^II^/Ni^IV^ redox manifold and C–C and C–heteroatom couplings mediated by **144-OTf**. Adapted from references [124,125].

An original ligand design strategy was employed to accomplish the C–H bond nickelation of arenes and alkanes induced by *N*-fluoro-2,4,6-trimethylpyridinium triflate (NFTPT; Scheme 35) [124,125]. The identity of the Ni^IV^ platforms **142a–c** and **144-X** was corroborated using NMR and XRD. The decisive role of triflate to assist the C–H to C–Ni^IV^ bond conversion was demonstrated through the isolation of **143a** when the triflate was replaced by tetrafluoroborate. A Ni^IV^-driven C–H bond nickelation was found to be the preferred pathway by computational means (vs. the competing Ni^III^-mediated path). Reaction of isolated **144-OTf** with external nucleophiles **146a-h** led to the C–Nu coupled products **146a–h**, thereby ascertaining its capacity to promote C–C and C–heteroatom bond forming processes.

**Scheme 36:**
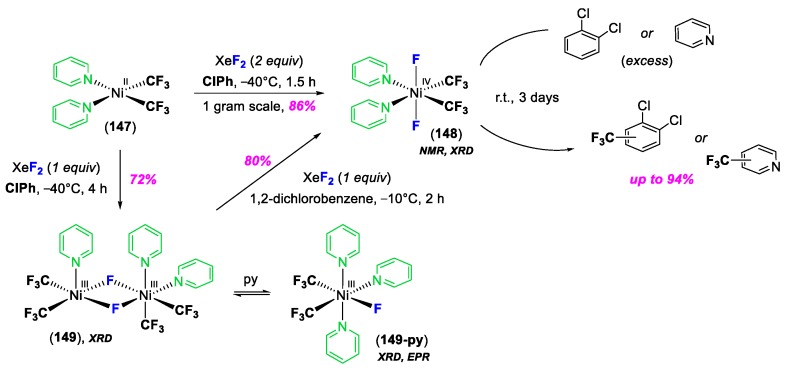
Synthesis of Ni^IV^-CF_3_ **148** and use in C(sp^2^)–H bond trifluoromethylation of arenes. Adapted from reference [126].

Our group has contributed to the field of C(sp^2^)–H bond functionalization and performed aromatic trifluoromethylations enabled by high-valent Ni^III^-CF_3_ and Ni^IV^-CF_3_ compounds named **149**, **149-py,** and **148**, respectively (Scheme 36) [126]. These high-valent species are: *i)* stabilized by simple, monodentate ligands (py, CF_3_, and F-itself); *ii)* easily accessible from identical sources (i.e., [(py)_2_Ni^II^(CF_3_)_2_] (**147**) and XeF_2_); *iii)* remarkably stable (isolable); and *iv)* authenticated using XRD and EPR (**149**, **149-py**) or XRD and NMR (**148**). Both Ni^III^-CF_3_ and Ni^IV^-CF_3_ species underwent the C–H bond breaking/C–CF_3_ bond forming sequence of arenes (1,2-dichlorobenzene or pyridine) with excellent yields (up to 94%) and intriguing selectivity.

**Scheme 37:**
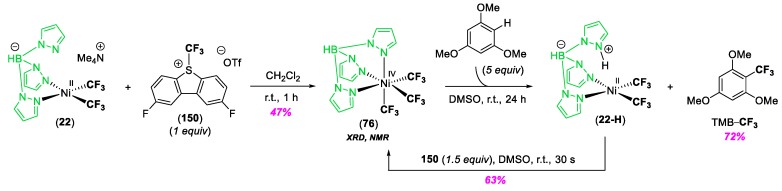
Synthesis of the catalytically efficient [(Tp)Ni^IV^(CF_3_)_3_] (**76**) via Ni^II^/Ni^IV^ redox manifold. Adapted from reference [127].

The group of Sanford has isolated and fully characterized the Ni^IV^-CF_3_ complex **76** (Scheme 37) [127], which is the first Ni^IV^ species bearing three CF_3_ groups. Assisted by the Tp ligand, **76** reacts in a stoichiometric fashion with 2,4,6-trimethoxybenzene (TMB) to yield the corresponding benzotrifluoride TMB–**CF_3_** and the Ni^II^-CF_3_ complex **22-H**, which was in situ re-oxidized to **76** by **150**. Most remarkably, **76** represents the first authenticated Ni^IV^ catalyst for the C(sp^2^)–H bond trifluoromethylation of (hetero)arenes (turnover number (TON) up to 5), including the industrially relevant scaffolds tadalafil, melatonin or *boc*-L-tryptophan [127]. Mechanistic studies supported the involvement of ^●^CF_3_ radicals and Ni^II^-CF_3_, Ni^III^-CF_3_ and Ni^IV^-CF_3_ intermediates.

**Scheme 38:**
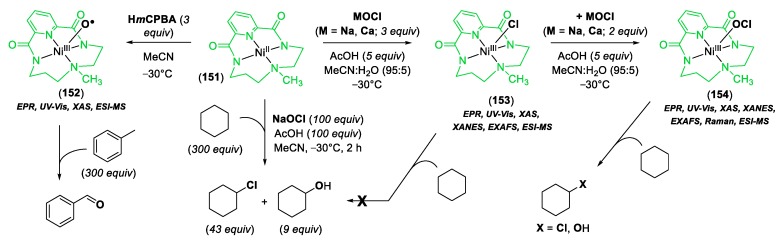
Synthesis, characterization and reactivity of high-valent Ni^III^ species **152**–**154**. Adapted from references [128,129].

Company and co-workers have synthesized the Ni^III^-oxyl complex **152 [128]** and the high-valent compounds Ni^III^-Cl **153** and Ni^III^-OCl **154** by reaction of the tetraaza-Ni^II^ precursor **151** and *meta*-chloroperbenzoic acid (H*m*CPBA) and CaOCl + acetic acid [129], respectively (Scheme 38). The structures of **152–154** were ascertained by exhaustive spectroscopic characterization and DFT-calculations. This work has demonstrated the ability of the Ni^III^-oxyl **152** and the Ni^III^-OCl **154** to promote C(sp^3^)–H bond oxidation of organic substrates.

**Scheme 39:**
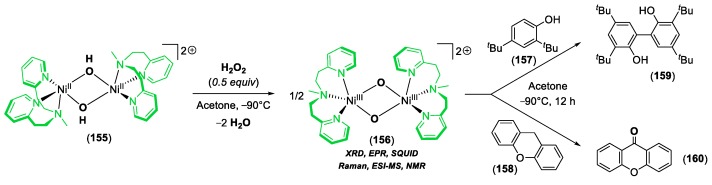
Synthesis and full characterization of the Ni^III^ dimer **156**, and its use to mediate C–H bond oxidation of phenols and activated alkanes. Adapted from reference [130].

A dinuclear Ni^III^ complex participating in C–H bond activation and the ensuing C–C or C=O bond forming reactions were published by Morimoto, Itoh and co-workers [130]. The Ni^III^ species **156** bearing the triazadentate ligand dpema was synthesized by treatment of the Ni^II^ precusor **155** with H_2_O_2_ in acetone at −90 °C (Scheme 39) [130]. Anion exchange with NaBPh_4_ permitted the selective crystallization of **156** that was appropriately characterized (XRD, EPR, magnetic measurements (Superconducting Quantum Interference Device (SQUID)), Raman, and ESI-MS). The keen analysis of **156** demonstrated the unprecedented triplet ground state for a high-valent [M_2_(*μ*-O)_2_] core, along with the ferromagnetic coupling of the Ni^III^ centers. In addition, **156** mediated the selective C–H bond functionalization of 2,4-di(tert-butyl)phenol (**157**) or xanthene (**158**) yielding **159** and **160**, respectively.

**Scheme 40:**
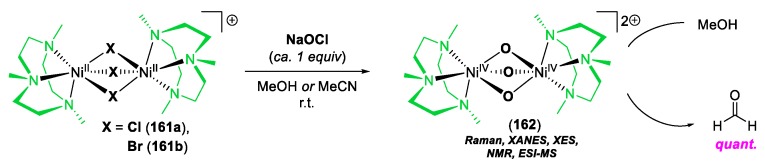
Synthesis of the dicationic [(Me_3_tacn)Ni^IV^(*μ*-O)_3_]^2+^ complex **162** and oxidation of methanol to formaldehyde enabled by **162**. Adapted from reference [131].

A dinuclear Ni^IV^ that mediates C–H bond functionalization was synthesized and characterized by Swart and Browne (Scheme 40) [131]. The complex [(Me_3_tacn)Ni^IV^(*μ*-O)_3_]^2+^ (**162**), attained from the dinuclear Ni^II^ complexes **161a,b** and NaOCl, represents a rare example of an isolated dinuclear Ni^IV^ complex. Its structure was determined by NMR, Raman spectroscopy (labelling experiments), XANES, XES, ESI-MS, and computational data. The C–H functionalization mediated by **162** proved viable for several substrates (methanol, xanthene, 9,10-dihydroanthracene, and fluorene).

## 5. Miscellaneous

Other interesting transformations dealing with high-valent Ni complexes that are involved in cross-coupling events and bond forming reactions are disclosed in this section. Hereafter, a short selection of cyclization reactions, C–heteroatom or N–N bond forging reactions, and olefin functionalization mediated by Ni^III^ or Ni^IV^ are collected.

**Scheme 41:**
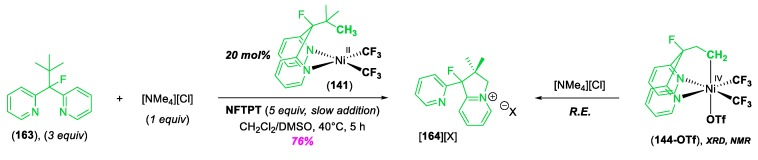
C(sp^3^)–H bond breaking/C(sp^3^)–N bond forming sequence via Ni^II^/Ni^IV^ catalysis. Synthesis of the heterocyclic salt [164][X]. Adapted from reference [125].

A first example is constituted by the **141**-catalyzed synthesis of heterocyclic salt [164][Cl] (Scheme 41) [125]. In this work, the functionalized bipyridine **163** was converted to [164][Cl] in 76% yield upon mild heating in presence of [NMe_4_][Cl], NFTPT (excess) and 20 mol% of Ni^II^-CF_3_ catalyst **141** (TON ca. 4). The viability of a Ni^II^/Ni^IV^ redox scenario was strongly supported by the stoichiometric reaction of **144-OTf** with chloride anions that provided the heterocyclic salt [164][X] (X= Cl, OTf) in nearly quantitative yield.

**Scheme 42:**
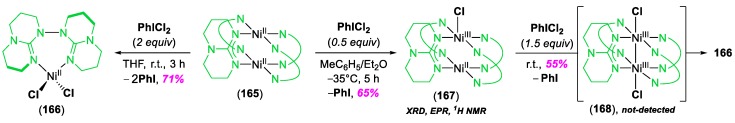
N–N bond formation from an assumed Ni^III^–Ni^III^ species **168**, generated from the mixed valence compound **167** and PhICl_2_. Adapted from reference [132].

Diao and co-workers discovered the N–N coupling of the guanidine derivative triazabicyclodecene (TBD) starting from the Ni^II^ complex **165** and PhICl_2_ (Scheme 42) [132]. Addition of PhICl_2_ (0.5 equivalents) to **165** at low temperature allowed the isolation and characterization of the Ni^II^-Ni^III^-Cl mixed valence compound **167**. The *trans*-influence of the chloride ligand in **167** prevented Ni–Ni bond interactions giving rise to a rare Ni^II^-Ni^III^-Cl homobimetallic complex with a zero order Ni–Ni bond. The isolated material **167** resulted to be coupling inactive. In the presence of PhICl_2_, **167** underwent instantaneous N–N bond formation involving an elusive Cl-Ni^III^-Ni^III^-Cl species **168**, which is reminiscent of Ritter’s Pd^III^ chemistry [98].

**Scheme 43:**
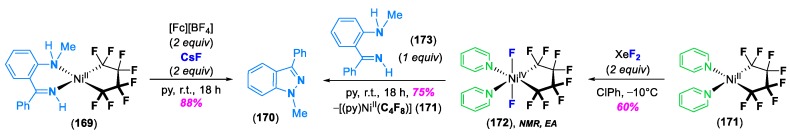
Isolable Ni^IV^F_2_ **172** and its capacity to promote N–N bond forming processes. Adapted from reference [133].

An indazole scaffold was synthesized in high yield by Vicic and co-workers from the perfluorinated metallacycle **169** in presence of mild oxidant, base and a fluoride source (Scheme 43) [133]. Once isolated and conveniently characterized (NMR and EA), the isolated Ni^IV^F_2_ **172** underwent N–N cyclizative coupling upon addition of **173** and pyridine to build **170**. The formation of **170** requires: *i)* coordination of **173** to **172**; *ii)* deprotonation of the N–H moiety ligated to Ni^IV^; and *iii)* R.E. step and recovery of the Ni^II^(C_4_F_8_) fragment.

**Scheme 44:**
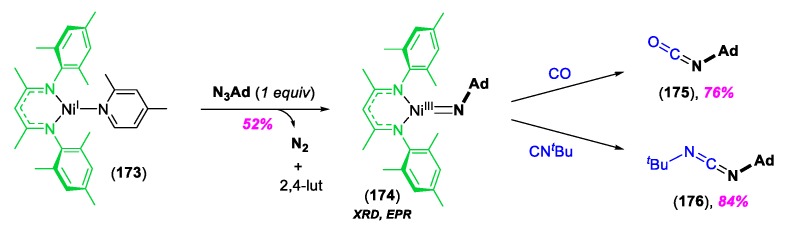
Imido-group transfer reactions mediated by the Ni^III^=NAd complex **174**. Adapted from reference [134].

**Scheme 45:**
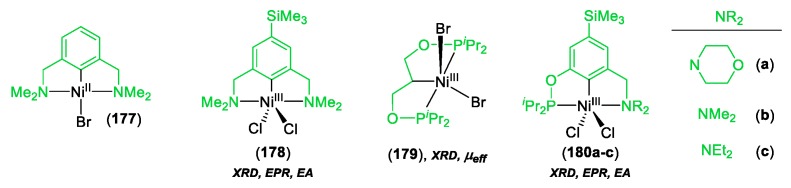
Ni-pincer complexes involved in Kharasch addition reactions [135,136,137,138,139,140,141,142,143].

The imido transfer reaction from M=NR fragments to organic substrates represents an innovative approach to build C–N bonds. In this sense, Warren disclosed the synthesis of the Ni^III^-imido complex **174** by reacting the Ni^I^ precursor **173** with adamantylazide (AdN_3_ in Scheme 44) [134]. The Ni^III^=NAd complex was isolated in 52% yield and studied using XRD, EPR, and DFT-calculations. The imido-group transfer from **174** proved viable towards CO and CN*^t^*Bu yielding cumulenes **175** and **176** in high yields.

van Koten performed Kharasch additions involving aryl-Ni^III^ pincer complexes allowing for the double functionalization of olefins [135,136,137,138,139,140,141]. Mechanistic studies (IR, EPR, and NMR) using the aryl-Ni^II^ **177** pointed to the participation of mononuclear aryl-Ni^III^ intermediates (Scheme 45) [135,136,137,138,139,140]. The C–halogen bond scission and concomitant Ni^II^ to Ni^III^ oxidation was found to be the rate determining step of the catalytic cycle [138]. Later, the same group isolated and characterized (XRD, EPR, and EA) the aryl-Ni^III^Cl_2_ species **178** by reacting the corresponding aryl-Ni^II^-Cl complex and CCl_4_, thus proving right their initial hypothesis [140]. Zargarian isolated the catalytically active Ni^III^X_2_ complexes **179** and **180a–c** bearing bis(phosphinite) (POCOP) or phosphinite-amine (POCN) based pincer-type ligands (Scheme 45) [141,142,143]. The novel Ni^III^ platforms were authenticated using diverse techniques (including XRD), and mediated catalytic Kharasch additions.

## 6. Summary and Conclusions

The study of fundamental organonickel chemistry and the use of nickel complexes in organometallic catalysis represent a jointly emerging research field. The main reasons are: *i)* the higher abundance and lower price of Ni compared with 4d and 5d metals; *ii)* the very rich and diverse redox reactivity of organonickel compounds; and *iii)* its enhanced reactivity that provides more room for reaction discovery. However, the air and moisture sensitivity of Ni-compounds and their propensity to undergo single electron transfer (SET) processes makes the mechanistic elucidation more challenging. This is particularly true for the commonly invoked, yet rarely proved, involvement of high-valent organonickel species in catalytic reaction mechanisms, including the highly demanded cross-coupling reactions. Here, the appropriate design of the ancillary ligand plays a pivotal role in improving the stability of Ni^III^ and Ni^IV^ complexes, thus allowing for their characterization and the discovery of their unprecedented reactivity. In this sense, this review aims to provide a general overview of most common strategies to successfully stabilize coupling active high-valent Ni species, namely: *i)* the coordination of polydentate *N*-donor ligands (Tp, Py_3_CH, pyridinophane derivatives…); *ii)* the incorporation of nickelacyclic cores; or *iii)* the use of strong *σ*-donating perfluorinated ligands (CF_3_ or the C_4_F_8_ fragment).

On the other hand, most representative work enclosed in the field of cross-coupling reactions enabled by spectroscopically characterized Ni^III^ and Ni^IV^ compounds are herein disclosed [144,145]. As a representative example, while low-valent Ni catalysts perform well for classical cross-coupling events, the intermediacy of high-valent Ni compounds becomes necessary in order to achieve more challenging transformations such as the C–F or C–CF_3_ bond-forming reactions. In addition to their enhanced activity, distinct reactivity patterns are displayed quite frequently by high-valent organometallic compounds. This was perfectly illustrated by the efficient C(sp^3^)–heteroatom coupling found for the Ni^IV^ platforms **50** and **52** instead the more favorable C(sp^2^)–heteroatom bond formation, commonly mediated by low-valent Ni compounds.

The study and deep understanding of the elementary reactions occurring for the high oxidation states of Ni^III^ or Ni^IV^ permitted to broaden the scope of transformations enabled by Ni^III^ and Ni^IV^ species. The current State of the Art for Ni^III^ and Ni^IV^ mediated bond forming reactions includes: *i)* C–C and C–heteroatom bond formation; *ii)* C–H bond functionalization; and *iii)* alternative N–N and C–heteroatom couplings. Most remarkably, the first two approaches to Ni^II^/Ni^IV^ catalysis have been reported recently and allowed for the C–H bond trifluoromethylation of industrially-relevant (hetero)arenes and C–N cyclization reactions. With no doubt, future work will expand the array of transformations mediated by Ni^III^ and Ni^IV^ species, and more catalytic applications mediated by a Ni^II^/Ni^IV^ redox scenario will appear soon.
